# Determination of patients requiring elective surgery following successful endoscopic detorsion in sigmoid volvulus

**DOI:** 10.12669/pjms.336.13921

**Published:** 2017

**Authors:** Sabri Selcuk Atamanalp, Refik Selim Atamanalp

**Affiliations:** 1 Prof. Sabri Selcuk Atamanalp, MD, Department of General Surgery Faculty of Medicine, Ataturk University, Erzurum, Turkey; 2 Refik Selim Atamanalp, English Medicine Program Faculty of Medicine, Ataturk University, Erzurum, Turkey

**Keywords:** Sigmoid Colon, Volvulus, Endoscopic Detorsion, Elective Surgery

## Abstract

Sigmoid volvulus (SV) is an unusual type of colonic obstruction, in which the sigmoid colon wraps around itself. As a rule, SV is decompressed via endoscopy in uncomplicated cases, while surgery is required in complicated cases. However, the recurrence rate following endoscopic detorsion is relatively high, with a relatively high mortality rate. Consequently, as a prophylactic procedure to prevent recurrence, elective surgery is suggested in some patients. Nevertheless, the selection criteria are open to questioning. In this report, we evaluate those criteria based on the broad experience of our clinic, and suggest elective surgery in patients in ASA classes I-III.

## INTRODUCTION

The principal treatment strategy in sigmoid volvulus (SV) is emergency endoscopic detorsion in uncomplicated patients, while emergency surgery is recommended for patients with sigmoid gangrene, sigmoid perforation, peritonitis, or unsuccessful endoscopic detorsion.^1^,^2^ Although the success rate of endoscopic detorsion is high (60-80%), the risk of SV recurrence following endoscopic detorsion is also high (30-90%) and carries a mortality risk of up to 35%.^3^-^6^ As a result, after successful endoscopic detorsion, elective sigmoid resection with primary anastomosis, particularly via a laparoscopic approach, is recommended in select patients.^7^,^8^ Nevertheless, the selection criteria for elective surgery are not well-discussed or identified in the literature.^9^,^10^

Although SV is relatively a rare disease, where we practice in Eastern Anatolia, it is an endemic area for SV.^11^,^12^ Our SV experience, which consists of 1,000 cases treated over a 51-year period between June 1966 and July 2017, comprises the largest single-center SV series in the world, according to major research databases, including Web of Science^9^ and PubMed.^10^ In this short report, we utilized this comprehensive clinical experience to identify the criteria for elective surgery for SV.

## CLINICAL EXPERIENCE AND INTERPRETATION

In our 1,000-patient series, of the 899 patients who provided information on anamnesis, 228 (25.36%) had a history of volvulus. The total SV count was 278 (once in 197 cases, twice in 18 cases, and three times or more in 13 cases). Non-operative detorsion was applied in 723 patients with a success rate of 82.35% and a mortality rate of 0.69%. Emergency surgery was used in 468 patients with a mortality rate of 16.45%. Elective surgery was performed in 111 patients with mortality and recurrence rates of 0.00%. The detailed data of our SV series are presented in Table-I. As shown, the expected mortality rate was 2.72% for our observed patients for the rest of their lives.

**Table-I:** Detailed data on our SV series.

*Parameter*	*Calculation*	*Result*
Recurrence rate	228/899	25.36%
Recurrence count	278	278
Total SV count	899+278	1,177
SV count for each patient	1,177/899	1.31
Mortality rate of nonoperative procedures	5/723	0.69%
Mortality rate of emergency surgery	77/468	16.45%
Total mortality rate for each SV	82/1,000	8.20%
Expected SV rate for observed patients	25.36%x1.31	33.22%
Expected mortality rate for observed patients	8.20%x33.22%	2.72%

The physical status classification system of the American Society of Anesthesiologists (ASA),^13^ which is shown in Table-II, is commonly used in the preoperative evaluation of patients. As seen, the mortality rates of patients in ASA class I or II are less than 0.4%, which is lower than that of our observed patients. In contrast, the mortality rates of patients in ASA class IV or V are more than 7.8%, which is higher than that of our observed patients. In SV, when we utilize the selection criteria for elective surgery based on the results of our series, the decision for the patients is simple; elective surgery can be easily suggested for patients in ASA class I or II, but should not be offered for those in ASA classes IV and V. However, the mortality rate of patients in ASA class III is 3.05% on average, which is comparable with the 2.72% mortality rate in our observed patients. Therefore, decisions regarding the elective surgery in patients in ASA class III is difficult and constitutes a major discussion point. In our experience, particularly in well-selected patients, the surgical mortality of elective procedures may be assumed to be zero (as it was in our series) or nearly zero. Consequently, we can easily recommend elective surgery, particularly via a laparoscopic approach, in most patients in ASA class III.^14^

**Table-II:** The physical status classification system of the ASA.^13^

*ASA score*	*Description*	*Mortality rate*
I	A normal patient (non-smoking, no or minimal alcohol use)	0.06-0.08%
II	A patient with mild systemic disease (current smoker, alcohol drinker, pregnancy, obesity BMI < 40, well-controlled diabetes mellitus, mild lung disease)	0.27-0.4%
III	A patient with severe systemic disease (active hepatitis, myocardial infarction > 3 months, implanted pacemaker, chronic obstructive pulmonary disease, diabetes mellitus, obesity BMI ≥ 40)	1.8-4.3%
IV	A patient with life-threatening systemic disease (myocardial infarction < 3 months, coronary artery disease/stent, cerebrovascular accident, transient ischemic attack, disseminated intravascular coagulation, renal failure, shock, sepsis)	7.8-23%
V	A moribund patient	9.4-51%

## DISCUSSION

In SV, as a clinical practice guideline, after resolution of the acute phase, Vogel et al.^1^ considered sigmoid colectomy to prevent recurrence as a strong recommendation, even if based on low-quality evidence. Kapadia^3^ reported the risk of recurrence after endoscopic detorsion can be as high as 90%, with a high mortality rate of up to 35%. Perrot et al.^4^ reported the recurrence rate of SV following endoscopic detorsion was 45-71%, with a mortality rate of 9-36%. Bruzzi et al.^5^ reported recurrence and mortality rates of 67% and 20%, respectively. Similarly, in our 453-case elderly patient series, we found recurrence and mortality rates of 30.7% and 12.4%, respectively.^15^ In a single study comparing treatment options according to the ASA scores of patients, Ifversen and Kjaer^6^ found a lower mortality rate and a longer survival duration in the electively treated group than that of the observed group, even if the median ASA score was lower in the elective surgery group (ASA II vs. ASA III). Based on this evidence, it is clear that recommending an elective surgery following a successful endoscopic detorsion is an acceptable treatment strategy for SV. Furthermore, considering that the recurrence and mortality rates of our series are lower than those reported above, we can easily recommend elective surgery in patients in ASA class III in addition to patients in classes I and II.

## CONCLUSION

In conclusion, after successful endoscopic detorsion, we recommend elective sigmoid resection with primary anastomosis, particularly via a laparoscopic approach, in well-prepared patients in ASA classes I-III. However, further studies, particularly prospective randomized trials, are needed to confirm our thesis, which is based on our broad experience with Sigmoid volvulus.

### Authors’ contribution

**SSA** designed the study, collected and analysed the data, reviewed the literature and prepared the manuscript

**RSA** reviewed the literature and edited the manuscript
